# Molecular Identification and Survey of *Tetratrichomonas buttreyi* and *Pentatrichomonas hominis* in Cattle in Shanxi Province, North China

**DOI:** 10.3390/ani15131899

**Published:** 2025-06-27

**Authors:** Yu-Xuan Wang, Tao Jia, Zi-Rui Wang, Jin-Long Wang, Ze-Dong Zhang, Ze-Xuan Wu, Wen-Wei Gao, Xing-Quan Zhu, Qing Liu

**Affiliations:** 1 Laboratory of Parasitic Diseases, College of Veterinary Medicine, Shanxi Agricultural University, Jinzhong 030801, China; wangyuxuan1113@126.com (Y.-X.W.); jt1178241595@163.com (T.J.); wangzirui8091@126.com (Z.-R.W.); jinlong1279@163.com (J.-L.W.); zhangzedong0519@126.com (Z.-D.Z.); wuzexuan0602@163.com (Z.-X.W.); sxndgaowenwei@163.com (W.-W.G.); 2 The Yunnan Key Laboratory of Veterinary Etiological Biology, College of Veterinary Medicine, Yunnan Agricultural University, Kunming 650201, China

**Keywords:** cattle, prevalence, sequence analysis, Shanxi Province, trichomonads

## Abstract

Several trichomonad species have already been reported from cattle, including *Tetratrichomonas buttreyi* and *Pentatrichomonas hominis*. However, there is currently a lack of information concerning the prevalence of trichomonad species in cattle in Shanxi Province, North China. In this study, 761 fecal samples from cattle across three counties in Shanxi Province, namely Qi, Jishan, and Shanyin, were examined for the presence of *T. buttreyi* and *P. hominis* DNA through a nested PCR assay targeting a specific segment of the small subunit ribosomal RNA (SSU rRNA) gene. The results showed that the total prevalence of *T. buttreyi* in cattle was found to be 74.5%, with region and sex identified as risk factors for infection. *P. hominis* exhibited an overall prevalence of 3.0%, with strong associations observed between infection and both region and age. Sequencing analysis indicated that some *T. buttreyi* isolates and all *P. hominis* isolates were identical to those reported previously based on the analysis of SSU rRNA sequences, while certain *T. buttreyi* isolates exhibited minor allelic variations. These results enhance our understanding of the geographical distribution and genetic characterization of *T. buttreyi* and *P. hominis* in cattle.

## Simple Summary

Cattle are natural hosts of *Tetratrichomonas buttreyi* and *Pentatrichomonas hominis*, and could be a potential source of *P. hominis* infections for other animals and humans. However, there have been no reports of *T. buttreyi* and *P. hominis* in cattle in Shanxi Province to date. In this study, 761 fecal samples were collected from cattle in Shanxi Province of China and screened for the two trichomonad species by nested PCR analysis of the small subunit ribosomal RNA (SSU rRNA) gene. Our results clearly demonstrated that the most common species was *T. buttreyi*, showing a total prevalence of 74.5%. Its prevalence was associated with region and sex. Only 23 samples tested positive for *P. hominis*, with a total prevalence of 3.0%. For *T. buttreyi*, eighteen distinct sequences were obtained, whereas only one representative sequence was obtained for *P. hominis*. To the best of our knowledge, the current study is the first to reveal the prevalence and perform genetic characterization of *T. buttreyi* and *P. hominis* in cattle in Shanxi Province, North China.

## 1. Introduction

Trichomonads are anaerobic flagellated protists that belong to the Parabasalia group, which comprises a few free-living species isolated from environmental samples [1,2]. However, most known trichomonads are endobiotic symbionts, commensals, or parasites of various vertebrate and invertebrate species [2,3]. The main trichomonad species reported in cattle are *Tritrichomonas foetus*, *Tetratrichomonas buttreyi*, and *Pentatrichomonas hominis* [3,4,5].

Although *T. foetus* is an important urogenital pathogen causing bovine trichomonosis [6], it was excluded in the present investigation due to this study’s focus on trichomonad species in the intestinal tract of cattle. *T. buttreyi* was initially discovered in the ceca and small intestine of pigs [7]. Subsequently, this organism was reported in ruminal and cecal contents of cattle [8]. Although *T. buttreyi* was found in the feces of a heifer, this trichomonad species was considered to be nonpathogenic to cattle [3,9]. However, more surveys of *T. buttreyi* in cattle with or without clinical symptoms will be required to reach a final conclusion. For *P. hominis*, previous studies have detected this trichomonad species in the fecal samples of both cats and dogs suffering from diarrhea, as well as in human patients [10,11,12]. Recently, the high prevalence of *P. hominis* (nine types were observed, and CCH1 was the predominant type compared with CCH4–11) in gastrointestinal cancer patients has been reported [13]. Using 16S rRNA high-throughput sequencing, a recent study preliminarily revealed that colon cancer patients infected with *P. hominis* exhibited specific changes in gut microbiota compared with uninfected colon cancer patients [14]. Recently, a survey was conducted in Anhui Province of China and concluded that cattle could be a potential source of *P. hominis* infection in humans and other animal hosts [15]. More investigations are needed to understand the roles of cattle in cross-species transmission, such as genotyping studies of *P. hominis.*

In addition to *T. foetus* in the urogenital tract of cattle, identification and genetic analysis of *T. buttreyi* and *P. hominis* in the intestinal tract of cattle based on larger sample sizes from more diverse sampling sites are also of importance. To date, however, there are a lack of sufficient data describing the prevalence and genetic characterization of the two intestinal trichomonad species in cattle worldwide. Specifically, only one survey was conducted in a province in South China [15]. Meanwhile, research data on the risk factors of *T. buttreyi* and *P. hominis* infection in cattle are limited. Hence, the PCR-based prevalence assessment and genetic analysis of *T. buttreyi* and *P. hominis* in cattle in Shanxi Province, North China, were carried out in the present study. Regarding risk factor assessment, region (Northern, Central, and Southern Shanxi; the temperature and humidity in this province increase from north to south), age, type of cattle, and sex were included in the present study.

## 2. Materials and Methods

### 2.1. Sampling Site Description and Sample Collection

Shanxi Province (latitude 34°34′–40°44′ N and longitude 110°14′–114°33′ E) is located on the Loess Plateau of China, the climate type of which is classified as a temperate continental monsoon climate zone. In November 2020, we collected 761 fecal samples from beef cattle (*n* = 376) and dairy cattle (*n* = 385) in the following counties: Jishan, Qi, and Shanyin. The counties chosen for this investigation are in the southern, central, and northern parts of Shanxi Province, respectively. Samples were collected using polyethylene (PE) gloves, with care to collect from the top layer of freshly voided feces to avoid environmental contamination. During the collection of fecal samples, the relevant information regarding age, sex, and geographical location for each animal was documented. All cattle used for sampling were clinically normal and without obvious signs of diarrhea. All samples were placed into a foam box filled with ice packs and transported to the laboratory. Once there, the fecal samples were stored at −20 °C until analysis.

### 2.2. DNA Extraction and PCR Amplification

Approximately 0.2 g of each fecal sample was subjected to genomic DNA extraction by using a commercial E.Z.N.A Stool DNA extraction kit (Omega Bio-tek Inc., Norcross, GA, USA) according to the manufacture-recommended procedures. The extracted DNA samples were subsequently kept at −20 °C until they were subjected to PCR as template DNA.

To determine the presence of *T. buttreyi* DNA, all the samples were tested using a species-specific nested PCR protocol to amplify a 623 bp fragment of the small subunit ribosomal RNA (SSU rRNA) gene. The PCR primers and amplification procedures referred to a previous study [16]. Briefly, the target DNA was firstly amplified by a first-round PCR (PCR1) using a pair of outer primers (forward: 5′-GCGCCTGAGAGATAGCGACTA-3′; reverse: 5′-GGACCTGTTATTGCTACCCTCTTC-3′) under the following thermal cycling conditions: an initial denaturation step (95 °C for 10 min); then 30 cycles comprising 1 min at 95 °C, 1 min at 60 °C, and 1 min at 72 °C; and a 10 min 72 °C final extension step. In a second round of PCR (PCR2), a new set of primers (forward: 5′-GTTTTTTCTCAGGCAGCAATG-3′; reverse: 5′-GCAACCTAGAAACCTAGGCG-3′) was used, following these thermal conditions: a 10 min initial incubation at 95 °C, followed by 30 cycles of 95 °C for 45 s, 60 °C for 45 s, and 72 °C for 45 s, and a final extension executed at 72 °C for 10 min.

Also, two rounds of PCR (species-specific primers were applied to the second round) were performed to determine the presence of *P. hominis* DNA. PCR1 was accomplished with the primers and cycling conditions identical to those of PCR1 used for the detection of *T. buttreyi* DNA. PCR2 was carried out using the oligonucleotides 5′-TGTAAACGATGCCGACAGAG-3′ as forward and 5′-CAACACTGAAGCCAATGCGAGG-3′ as reverse primers, and the cycle parameters for the reaction included a pre-denaturation step of 10 min at 95 °C, followed by 30 cycles of denaturation (95 °C, 30 s), primer annealing (55 °C, 30 s) and primer extension (72 °C, 30 s), plus 1 cycle of extension at 72 °C for 10 min.

The final volume of each PCR reaction mixture was 25 µL and contained 2 μL of genomic DNA (or 2 μL of the products of PCR1 for PCR2), 0.4 μM of each forward and reverse primer, 200 mM of each deoxyribonucleotide triphosphate (dNTP), 1.5 mM of MgCl_2_, 2.5 µL 10 × PCR buffer without Mg^2+^, and 0.625 U of Ex Taq polymerase (TaKaRa, Dalian, China). For quality control (to exclude false-positive and false-negative reactions) and to confirm the identity of a band, negative (reagent-grade water without DNA) and positive (verified DNA of *T. buttreyi* or *P. hominis* by sequencing) control samples were incorporated into all PCR runs. After amplification, the secondary PCR products were electrophoresed in 1.5% agarose gels and visualized by ethidium bromide staining to check for the presence of amplicons with the expected size.

### 2.3. Sequencing and Phylogenetic Analysis

Following agarose gel electrophoresis, the expected PCR bands were purified and sequenced in both directions using the Sanger sequencing method under a 3730XL automatic DNA sequencer (Applied Biosystems, Thermo Fisher Scientific, Waltham, MA, USA) from Sangon Biotech Co., Ltd. (Shanghai, China). The resulting sequences obtained from Sanger sequencing were assembled and edited using Chromas Pro v2.1.3 software, and the Basic Local Alignment Search Tool (BLAST) program (http://www.ncbi.nlm.nih.gov/blast/) (accessed on 5 March 2024) was used to compare the final nucleotide sequences found in this study with reference sequences available in the GenBank database. To show existing patterns of polymorphism, multiple sequence alignment of the sequences obtained in this study, along with reference sequences retrieved from the NCBI GenBank database (some sequences showing 100% identity with one of the sequences obtained in this study or from interesting isolates were selected), was performed using the BioEdit Sequence Alignment Editor version 7.7.1.0. The neighbor-joining (NJ) method was employed to construct phylogenetic trees using the MEGA 7 software (http://www.megasoftware.net/) (accessed on 29 March 2024), with the mean genetic distances being calculated by the Kimura parameter-2 model [17,18]. To evaluate the reliability of the nodes in the NJ trees, a bootstrap analysis with 1000 replicates was performed.

### 2.4. Statistical Analysis

SPSS 26.0 software (IBM, Chicago, IL, USA) was used for data analysis, and the relationship between prevalence and its risk factors was tested in a chi-square analysis. Odds ratios (ORs) and their corresponding 95% confidence intervals (CIs) were calculated to test the level of association. When the *p*-value was less than 0.05, the difference was considered to be statistically significant.

## 3. Results

### 3.1. PCR Detection of T. buttreyi and P. hominis

**Figure 1:**
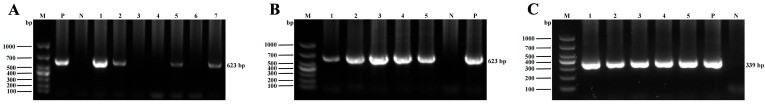
Representative PCR products were visualized by 1.5% agarose gel electrophoresis analysis. (**A**) Lane M: DNA molecular weight marker (100 bp); lane P: positive control; lane N: negative control; lanes 1–2, 5, and 7: dairy cattle fecal samples positive for *T. buttreyi*; lanes 3–4, and 6: dairy cattle fecal samples negative for *T. buttreyi*. (**B**) Lane M: DNA molecular weight marker (100 bp); lane P: positive control; lane N: negative control; lanes 1–5: beef cattle fecal samples positive for *T. buttreyi*. (**C**) Lane M: DNA molecular weight marker (100 bp); lane P: positive control; lane N: negative control; lanes 1–5: dairy cattle fecal samples positive for *P. hominis*.

As shown in Figure 1, samples that were positive for *T. buttreyi* and *P. hominis* showed a band of expected size for positive controls (approximately 623 bp and 339 bp, respectively), whereas all negative controls were free of amplicons.

### 3.2. Prevalence of T. buttreyi and P. hominis in Cattle in Shanxi Province

In the present study, a total of 567 *T. buttreyi*-positive samples were detected, with an overall prevalence of 74.5% (Table 1). At the county level, the highest prevalence was observed in Qi County (85.8%, 247/288), followed by Shanyin County (78.5%, 157/200) and Jishan County (59.7%, 163/273). There was a significant difference in the prevalence of *T. buttreyi* among the three counties (*p* < 0.001). The prevalence of *T. buttreyi* in female cattle (77.8%, 404/519) was higher than that in males (67.4%, 163/242), and a statistically significant difference between sex at the level of *p* = 0.002 was observed. For the two types of cattle, the prevalence of *T. buttreyi* in dairy cattle was 81.3% (313/385), which was significantly higher than that (67.6%, 254/376) in beef cattle (*p* < 0.001).

Of the 761 cattle fecal samples examined by nested PCR amplification of the SSU rRNA gene, 23 were found to be positive for *P. hominis*, with a total prevalence of 3.0% (Table 2). Among the three counties, the prevalence of *P. hominis* was 11.5% (23/200) in Shanyin, and we did not detect any positive samples in Qi and Jishan. Among age groups, *P. hominis* was only found in cattle younger than 12 months and older than 18 months, and the prevalence was 7.6% (21/278) and 0.7% (2/303), respectively. For the two types of cattle, *P. hominis* was only detected in dairy cattle, with a prevalence of 6.0% (23/385).

### 3.3. Sequence Alignment

**Figure 2:**
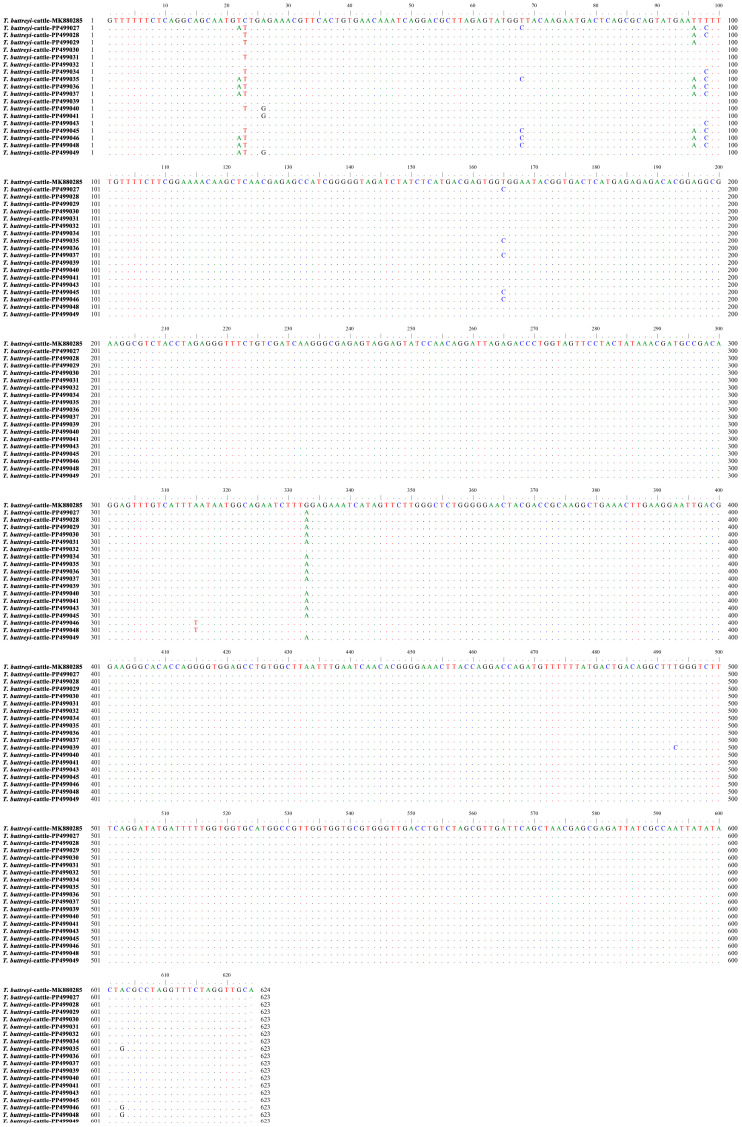
Sequences of the partial SSU rRNA gene from representative *T. buttreyi* isolates identified in the present study (PP499027–PP499032, PP499034–PP499037, PP499039–PP499041, PP499043, PP499045–PP499046, and PP499048–PP499049) were aligned against a reference sequence (MK880285). Dots indicate nucleotide bases that are identical to the consensus sequence, while a dash indicates that a nucleotide base was not included for analysis in the present study.

Sanger sequencing analysis of *T. buttreyi*-positive samples identified 18 distinct sequences, with sequence identities ranging from 98.4% to 99.8% (Figure 2). At present, the 18 distinct sequences are publicly available from the NCBI GenBank database (accession numbers: PP499027–PP499032, PP499034–PP499037, PP499039–PP499041, PP499043, PP499045–PP499046, and PP499048–PP499049). Among these representative sequences, 15 (PP499027, PP499030–PP499032, PP499034–PP499037, PP499040–PP499041, PP499043, PP499045–PP499046, and PP499048–PP499049) were found in beef cattle isolates, whereas nine (PP499027–PP499032, and PP499039–PP499041) were observed in dairy cattle isolates. One sequence (PP499032) showed a 100% similarity to those of *T. buttreyi* isolates derived from pigs in China (KX833156, PP256575, and KM205212), Germany (MK801506) and Philippines (JX565058), cattle in China (MK880285), and horses in the Czech Republic (AY886859). Meanwhile, one sequence (PP499039) showed a 100% similarity to those of *T. buttreyi* isolates reported earlier from pigs in the Philippines (JX565053), and Hanuman langurs (HQ149978) and wild boars (AY886865) in the Czech Republic. Of the remaining sixteen representative sequences, each shared high similarity to the sequence derived from cattle (MK880285) in China.

**Figure 3:**
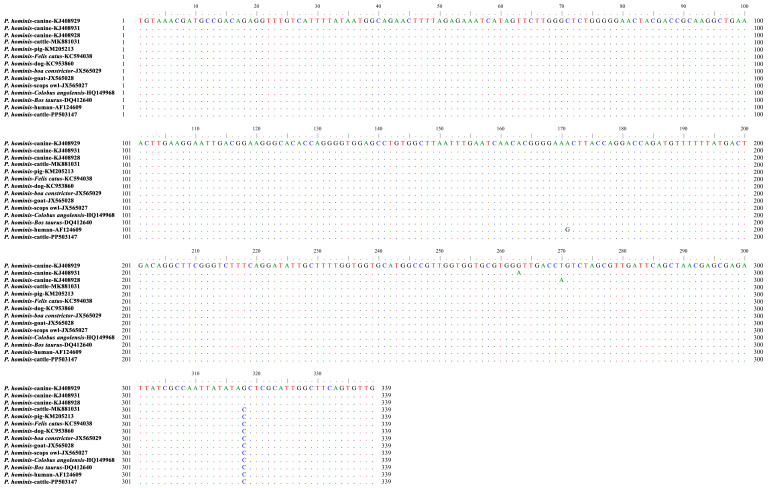
The representative sequence of *P. hominis* identified in the present study (PP503147) was aligned against reference sequences. Dots indicate nucleotide bases that are identical to the consensus sequence.

Of 23 *P. hominis*-positive samples, one representative sequence was identified. Sequence identity analysis showed that there was 100% homology of the sequence (PP503147) generated in the present study with those of *P. hominis* from different hosts, including cattle (MK881031 and DQ412640), macaques (MH997492), pigs (KX833161 and KM205213), dogs (OR033182 and KC953860), cats (OR033173 and KC594038), snakes (JX565029), goats (JX565028), scops owls (JX565027), and monkeys (HQ149968). The sequence did not belong to types CC1–3, and nucleotide 171 was A versus G in the sequence from a human (AF124609) (Figure 3).

### 3.4. Phylogenetic Relationships

**Figure 4:**
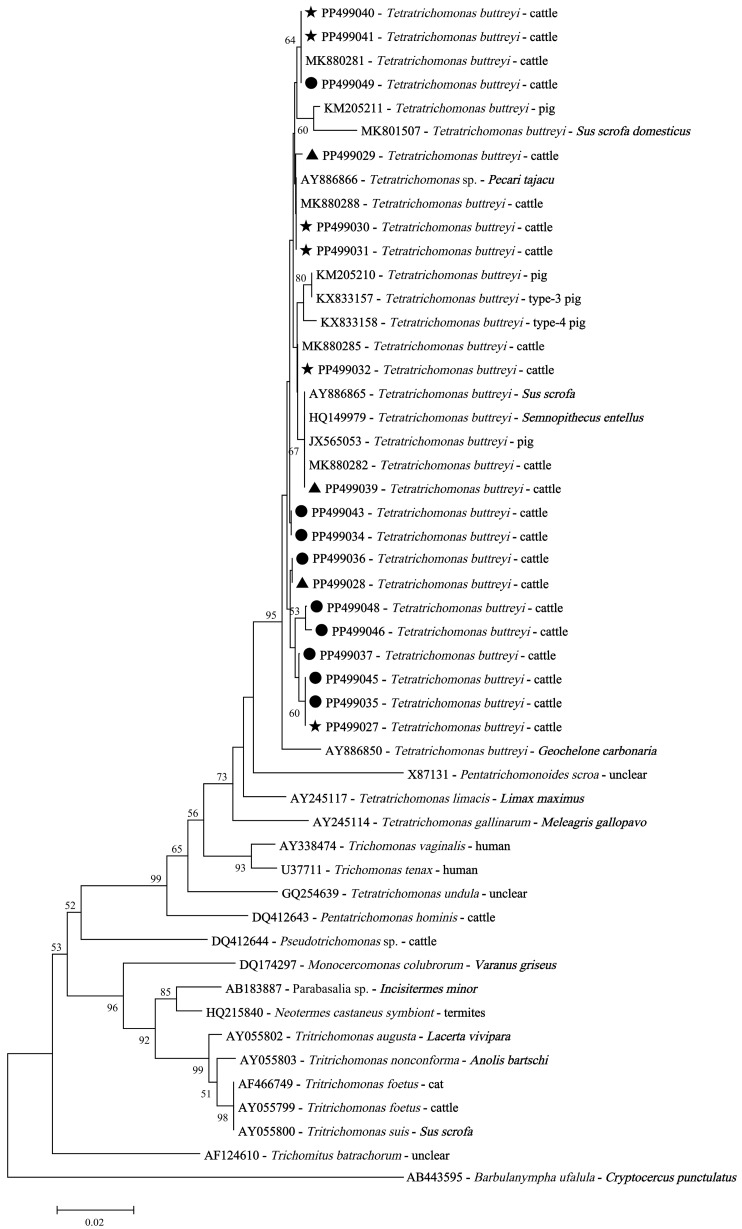
Phylogenetic relationships using neighbor-joining analysis among trichomonad isolates based on the SSU rRNA nucleotide sequences. Black triangles: the representative SSU rRNA sequences of *T. buttreyi* isolates derived from dairy cattle in this study; black circles: the representative SSU rRNA sequences of *T. buttreyi* isolates derived from beef cattle in this study; black pentagrams: the representative SSU rRNA sequences of *T. buttreyi* detected in both dairy and beef cattle isolates.

Phylogenetic analysis revealed genetic diversity in the *T. buttreyi* isolates derived from cattle (Figure 4). Although the sixteen representative sequences obtained in this study did not share 100% identity with relevant sequences available in GenBank, the phylogenetic analysis confirmed undoubtedly that all of these isolates belonged to *T. buttreyi* (Figure 4).

**Figure 5:**
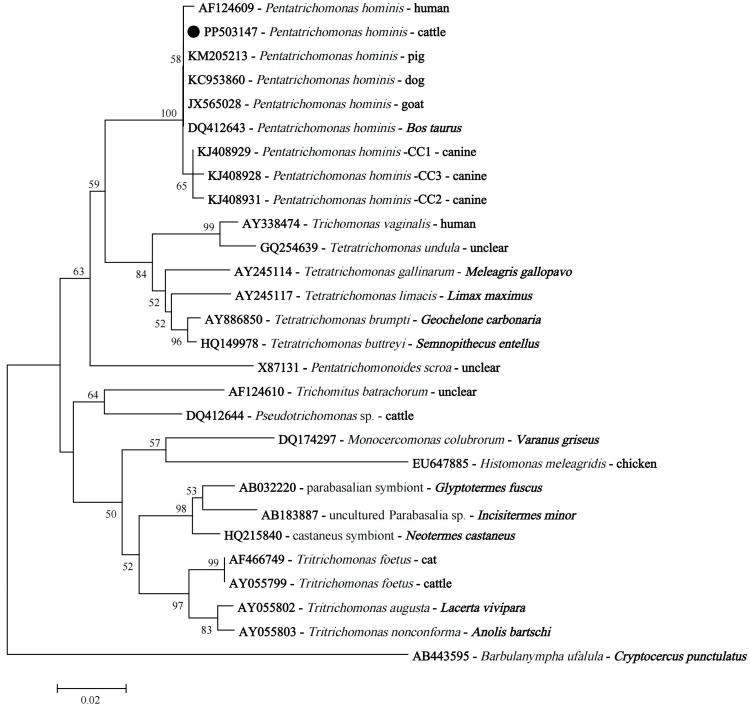
The neighbor-joining phylogenetic tree based on the SSU rRNA gene sequences shows the position of *P. hominis* isolates obtained in this study. The black circle indicates the representative SSU rRNA sequence of *P. hominis* isolates derived from cattle in this study.

Though there were differences in the SSU rRNA nucleotide sequences, phylogenetic analysis revealed that *P. hominis* isolates obtained in this study, *P. hominis* with sequences belonging to types CC1–3, and a human isolate had a close relationship (Figure 5).

## 4. Discussion

Due to the limited range of morphological features among different trichomonad species, it is difficult to distinguish *P. hominis* from other trichomonad species (such as *T. foetus*) through microscopic examination [10,19]. Molecular diagnostic methods have been introduced recently for species-level identification of trichomonad species. For example, a previous study showed that the definitive diagnosis of *T. foetus* infection in cats with clinical signs of diarrhea can be accomplished through the single-tube nested PCR [20]; species-level identification of *T. buttreyi*, *T. foetus*, and *P. hominis* can be achieved by SSU rRNA gene amplification using the species-specific primers [16]. Hence, in this study, we conducted prevalence assessment and genetic analysis of *P. hominis* and *T. buttreyi* in cattle in Shanxi Province based on amplification of the partial SSU rRNA gene by nested PCR and subsequent sequence analysis referring to a previous study [16]. This is the first study to provide molecular evidence on the presence of *P. hominis* and *T. buttreyi* in cattle in Shanxi Province, North China.

To date, there are knowledge gaps concerning the prevalence of *T. buttreyi* in cattle in China. Actually, only one previous study has investigated the prevalence of *T. buttreyi* in cattle in one Chinese province, Anhui [15]. Overall, the prevalence of *T. buttreyi* in cattle detected in this study was 74.5%, which was higher than the prevalence of 8.1% reported in Anhui Province [18]. Meanwhile, variations in prevalence among different regions were observed in the present study. This may be attributed to several factors, such as feeding and management practices. Due to the high prevalence of *T. buttreyi*, and to further analyze its pathogenicity, there is a need for continuous monitoring of the health status of cattle in the regions investigated in this study. Statistical analysis implied that region but not age was significantly associated with the prevalence of *T. buttreyi* in cattle in Shanxi Province, which was consistent with that reported by Li et al. [15]. Also, an investigation on the prevalence of *T. buttreyi* in raccoon dogs showed that age has no statistically significant association with *T. buttreyi* infection [21]. However, in a previous study, age was identified to be a risk factor for the prevalence of *T. buttreyi* in pigs [22]. Hence, further studies are needed to clarify the relationship between age and *T. buttreyi* infection. Dairy cattle had a significantly higher prevalence rate of *T. buttreyi* than beef cattle. We speculated that the difference between the prevalence rates may be related to different production systems for dairy and beef cattle rather than to breed differences, because a high prevalence was observed in both. In addition, sex as a risk factor for *T. buttreyi* infection is reported here for the first time. Certainly, more studies are needed before drawing a final conclusion.

The overall prevalence of *P. hominis* observed in the present study was 3.0%, which was similar to that reported for cattle in Anhui Province (5.4%) [15], but higher than that reported for goats (0.3%) and sheep (0.0%) in the central eastern region of China [23]. The differences in prevalence may be associated with fecal characteristics, as mentioned in a previous study [23]. The previous study also postulated that sheep may not be the natural hosts for *P. hominis*. Notably, while the prevalence of *P. hominis* in pigs in Jilin Province (a large province in the middle of Northeast China) was 24.1%, all 362 fecal samples collected from pigs in Shanxi Province were negative for *P. hominis* in our previous investigation [16,22]. Also, no beef cattle fecal samples were tested positive for *P. hominis* in the present study. Given that there are limited data regarding the prevalence of *P. hominis* in goats, sheep, and beef cattle, we thought that more investigations are needed to confirm the low prevalence of *P. hominis* in goats before analyzing the reasons accounting for the low prevalence and to answer the unresolved question of whether sheep and beef cattle act as natural hosts for *P. hominis*. A previous study showed that sex is a contributing risk factor for *P. hominis* infection in non-human primates [24], which was inconsistent with our results. The variations may be attributed to the combined effects of multiple factors.

For *T. buttreyi*, we found that two distinct sequences generated in this study exhibited 100% homology with those of *T. buttreyi* isolates reported earlier from different animals and from different geographical regions. Also, our study showed that the representative sequence derived from *P. hominis* had 100% identity with those of *P. hominis* isolates from different animals, regardless of geographic origin. This indicated the strong clonality and good genetic fitness of these isolates. Previous epidemiological studies also reported three types of nucleotide sequences of the SSU rRNA gene of *P. hominis*, namely CC1, CC2, and CC3 [25]. Of these, *P. hominis* with sequences belonging to type CC1 may have zoonotic potential, because type CC1 has been found in *P. hominis* isolates from humans, dogs, monkeys, goats, tigers, and foxes [23,26,27,28]. Conversely, the host ranges of *P. hominis* with sequences belonging to types CC2 and CC3 were limited, with no confirmed cases of zoonosis. The representative sequence of *P. hominis* isolates obtained in the present study did not belong to types CC1–3. Intriguingly, a previous investigation of *P. hominis* infection in cattle in Anhui Province also revealed that the representative sequence differed at 1–2 nucleotides from types CC1–3 [15]. Nevertheless, the representative sequences of *P. hominis* isolates reported in cattle were limited; in addition, phylogenetic analysis revealed that *P. hominis* isolates obtained in this study had a close relationship with a human isolate, as well as *P. hominis* having sequences belonging to type CC1. Hence, more studies are needed to better understand the genetic characterization and zoonotic potential of *P. hominis* in cattle. In addition, we observed minor allelic variations in the SSU rRNA sequences of other *T. buttreyi* isolates generated in the present study, providing new evidence in support of a high level of genetic similarity among *T. buttreyi* isolates.

## 5. Conclusions

The present study is the first describing the prevalence of *T. buttreyi* and *P. hominis* in cattle in Shanxi Province, North China, by employing a molecular technique. The results showed that cattle in this province had a low prevalence of *P. hominis*, whereas extensive prevalence of *T. buttreyi* in cattle was observed. Two representative sequences of *T. buttreyi* generated in this study were identical to the reference sequences previously deposited in GenBank, and sixteen novel sequences were found. For *P. hominis*, only one known sequence, previously reported in various host species, was found in this work. These findings broaden our understanding of the distribution and genetic information of the two trichomonad species in cattle in China.

## Figures and Tables

**Table 1:** Prevalence and factors associated with *Tetratrichomonas buttreyi* in cattle in Shanxi Province, North China.

Factor	Category	No. Examined	No. Positive	Prevalence % (95% CI)	OR (95%)	*p*-Value
Region	Qi Country	288	247	85.8 (81.7–89.8)	4.1 (2.7–6.1)	<0.001
	Jishan Country	273	163	59.7 (53.9–65.5)	Reference	
	Shanyin Country	200	157	78.5 (72.8–84.2)	2.5 (1.6–3.7)	
Age	Month < 12	278	216	77.7 (72.8–82.6)	1.5 (1.0–2.3)	0.141
	12 ≤ Month ≤ 18	180	125	69.4 (62.7–76.2)	Reference	
	Month > 18	303	226	74.6 (69.7–79.5)	1.3 (0.8–1.9)	
Sex	Male	242	163	67.4 (61.5–73.3)	Reference	0.002
	Female	519	404	77.8 (74.3–81.4)	1.7 (1.2–2.4)	
Type	Dairy cattle	385	313	81.3 (77.4–85.2)	2.1 (1.5–2.9)	<0.001
	Beef cattle	376	254	67.6 (62.8–72.3)	Reference	
Total		761	567	74.5 (71.4–77.6)		

**Table 2:** Prevalence and factors associated with *Pentatrichomonas hominis* in cattle in Shanxi Province, North China.

Factor	Category	No. Examined	No. Positive	Prevalence % (95% CI)	OR (95%)	*p*-Value
Region	Qi Country	288	0	0	-	<0.001
	Jishan Country	273	0	0	-	
	Shanyin Country	200	23	11.5 (7.1–15.9)	-	
Age	Month < 12	278	21	7.6 (4.5–10.7)	12.3 (2.9–52.9)	<0.001
	12 ≤ Month ≤ 18	180	0	0	-	
	Month > 18	303	2	0.7 (−0.3–1.6)	1	
Sex	Male	242	8	3.3 (1.1–5.6)	1.1 (0.5–2.7)	0.755
	Female	519	15	2.9 (1.5–4.3)	1	
Type	Dairy cattle	385	23	6.0 (3.6–8.3)	-	<0.001
	Beef cattle	376	0	0	-	
Total		761	23	3.0 (1.8–4.2)		

## Data Availability

The data sets supporting the results of this article have been submitted to GenBank, and the accession number is shown in the article.
